# Different and overlapping functions of *Arabidopsis* LHT6 and AAP1 transporters in root amino acid uptake

**DOI:** 10.1093/jxb/eru278

**Published:** 2014-07-08

**Authors:** Molly Perchlik, Justin Foster, Mechthild Tegeder

**Affiliations:** School of Biological Sciences, Washington State University, Pullman, WA 99164, USA

**Keywords:** AAP1, amino acid transporter, cellular import of organic nitrogen, LHT6, regulation, root nitrogen uptake, soil nitrogen acquisition, symplasmic and apoplasmic transport.

## Abstract

Plants acquire nitrogen in the form of amino acids from the soil, and transport proteins located in the plasma membrane of root cells are required for this process. It was found that the *Arabidopsis* lysine-histidine-like transporter LHT6 is expressed in root cells important for amino acid uptake, including the epidermis, root hairs, and cortex. Transport studies with *lht6* mutants using high levels of amino acids demonstrated that LHT6 is in fact involved in amino acid uptake. To determine if LHT6 plays a role in nitrogen acquisition at soil amino acid concentrations, growth and uptake studies were performed with low levels of toxic amino acid analogues and radiolabelled amino acids, respectively. In addition, mutants of AAP1, another root amino acid transporter, and *lht6*/*aap1* double mutants were examined. The results showed that LHT6 is involved in uptake of acidic amino acids, glutamine and alanine, and probably phenylalanine. LHT6 seems not to transport basic or other neutral amino acids, or, alternatively, other transporters might compensate for eliminated LHT6 function. Previous studies suggested that AAP1 only takes up amino acids at high concentrations; however, here it is demonstrated that the transporter functions in acquisition of glutamate and neutral amino acids when present at soil concentrations. When comparing the characterized root uptake systems, it appears that transporters both with overlapping substrate specificity and with preference for specific substrates are required to access the soil amino acid pool.

## Summary

Plants use soil amino acids as a nitrogen source. Plasma membrane-localized transport proteins are essential for the import of a broad spectrum of amino acids into the root cells.

## Introduction

Nitrogen (N) is needed by the plant in relatively high amounts to ensure sufficient growth, development, and reproduction. In agricultural systems, high biomass and seed production is often achieved through the supply of the inorganic N forms nitrate and ammonium, which can both be taken up by the root cells through the activity of plasma membrane transport proteins (Huang *et al.*, 1999; Kaiser *et al.*, 2002; Sohlenkamp *et al.*, 2002; Orsel *et al.*, 2007). However, in organic agriculture or other cropping systems that rely on degradation of organic matter such as manure for plant N nutrition, amino acids may represent an essential N source (Khan, 1971; Senwo and Tabatabai, 1998). Similarly, soils of some ecosystems contain significant amounts of amino acids, which may be the predominant N forms acquired by the root (Lipson *et al.*, 2001; Jones and Kielland, 2002; Näsholm *et al.*, 2009). While the preference of plants for inorganic (i.e. nitrate and ammonium) versus organic (i.e. amino acids) N forms has not been fully resolved, and might depend on the plant species (Streeter *et al.*, 2000; Näsholm and Persson, 2001; Thornton, 2001; Persson and Näsholm, 2002; Biernath *et al.*, 2008; Jamtgard *et al.*, 2008; El-Naggar *et al.*, 2009; Ge *et al.*, 2009), soil amino acid concentrations (Näsholm and Persson, 2001), and soil pH (Kielland, 1994; Jones and Kielland, 2002), it has been shown that amino acids are an important N source for plants even in the presence of nitrate and ammonium (Aslam *et al.*, 2001; Näsholm *et al.*, 2001; Henry and Jeffereies, 2003; Gioseffi *et al.*, 2012; Vinall *et al.*, 2012).

Root epidermal cells and root hairs comprise the majority of root surface area and are thought to be the main sites for uptake of nutrients, including amino acids, from the soil (Dittmer, 1937; Itoh and Barber, 1983; Lazof *et al.*, 1992, 1996; Gassmann and Schroeder, 1994; Gahoonia and Nielsen, 1998). Following entry into these root cells, amino acids move symplasmically to the xylem, which delivers the N to the shoot (Miller, 1985; Rentsch *et al.*, 2007; Tegeder and Rentsch, 2010). In addition, root-cap cells located at the root tip might import some organic soil N, and/or amino acids may move apoplasmically in the cell wall space of the root cortex until they reach the Casparian strip of the root endodermis where they must be imported into the symplast for partitioning to the vasculature (Miller, 1985; Barlow, 2002; Walch-Liu *et al.*, 2006). Uptake of amino acids into root hairs, epidermis, or along the apoplasmic route into the cortex and endodermis requires transport proteins mediating passage of the organic N across the plasma membrane (Tegeder, 2014).

Free amino acid concentrations in soils range from 0 to 150 μM, and while many of the protein amino acids are found, glutamate, aspartate, glutamine, asparagine, glycine, serine, and alanine are often predominant (Senwo and Tabatabai, 1998; Raab *et al.*, 1999; Schmidt and Stewart, 1999; Weigelt *et al.*, 2005). The type of transporter that is functioning in root uptake would depend on which amino acids are present in the respective rhizosphere and their concentrations. In *Arabidopsis*, to date three transport proteins have been demonstrated to affect root uptake of amino acids, and these are the amino acid permeases AAP1 (Lee *et al.*, 2007) and AAP5 (Svennerstam *et al*., 2008, 2011), and the lysine-histidine-type transporter LHT1 (Hirner *et al.*, 2006; Svennerstam *et al*., 2007, 2008, 2011). In addition, the compatible solute transporter ProT2 is involved in proline acquisition (Lehmann *et al.*, 2011). Of these amino acid transporters, only AAP1 has been shown to be localized to the root epidermis and root hairs (and root tip), where uptake of large amounts of nutrients might occur (Dittmer, 1937; Itoh and Barber, 1983; Lazof *et al.*, 1992, 1996; Gassmann and Schroeder, 1994; Gahoonia and Nielsen 1998), and to function in import of neutral and acidic amino acids (Lee *et al.*, 2007). Transport studies using mutants suggest that AAP1 might operate in soil N uptake at high amino acid concentrations (Lee *et al.*, 2007; Svennerstam *et al.*, 2011). In contrast, LHT1 and AAP5 seem to be involved in acquisition of neutral and acidic amino acids, and basic amino acids, respectively, at soil solution levels below 50 μM (Svennerstam *et al*., 2008, 2011). However, in young, developing *Arabidopsis* plants, LHT1 function in amino N uptake seems to be restricted to the root tips, as indicated by promoter–β-glucuronidase (GUS) studies (Hirner *et al.*, 2006), and in seedlings it is expressed throughout the root with the exception of root tips (Liu *et al.*, 2010). Localization of AAP5 within the root has not been resolved yet, although transcripts may be present in the root cortex and endodermis (Brady *et al.*, 2007; Gifford *et al.*, 2008).

Here, it is hypothesized that besides AAP1, which is involved in import of glutamate and neutral amino acids into the root hairs and epidermis when present at relatively high soil concentrations (Lee *et al.*, 2007; Svennerstam *et al.*, 2011), at least one additional transporter is functioning in the same cell types to access the broad spectrum of soil amino acids present at lower, naturally occurring concentrations during *Arabidopsis* development. Candidates might belong to the LHT family that is predicted to contain high affinity amino acid transporters for neutral and acidic amino acids (Chen and Bush, 1997; Lee and Tegeder, 2004; Hirner *et al.*, 2006). It was found that *LHT6* (At3g01760) is strongly expressed in roots and this transporter was examined further with respect to its function in amino acid root uptake using localization analyses, as well as growth and transport studies with *lht6* mutants. In addition, *aap1* and *lht6/aap1* double mutants were examined to analyse the potential cooperation of LHT6 and AAP1 in amino acid uptake.

## Materials and methods

### Plant materials and growth conditions

*Arabidopsis* (ecotype Columbia) wild-type and mutant plants were grown in 0.785cm^3^ pots containing Sungro professional growing mix LC1 (Seba Beach, AB, Canada) consisting of peat (70–80%), perlite, and domestic limestone (20–30%). Plant growth conditions were set to 16h light at 150–200 μmol photons m^–2^ s^–1^, 40% humidity, and day and night temperatures of 20 °C and 16 °C, respectively. For *in vitro* studies, *Arabidopsis* seeds were rinsed in 70% (w/w) ethanol followed by sterilization for 5min in a solution containing 0.5% SDS (w/v) and 2% NaOCl (w/v). The sterile seeds were stratified in water for 3 d at 4 °C, and then transferred to Petri dishes with solid growth medium (see below). Seeds and growing plants were cultured in environmentally controlled chambers at 16h light, 125–130 μmol photons m^–2^ s^–1^, 50% humidity, and a constant temperature of 20 °C.

### RNA expression analysis

Total RNA was isolated from 6-day-old seedlings as well as from roots, rosette leaves, source leaves, sink leaves, stems, buds, flowers, and siliques of 6-week-old plants grown on soil according to Pélissier and Tegeder (2007). First-strand cDNA synthesis was carried out using M-MLV reverse transcriptase (Invitrogen, Carlsbad, CA, USA) and oligo d(T) primers. PCR was performed with the cDNAs and gene-specific primers to resolve expression of *LHT6* in the different organs (5′-CTTAAGTGCACTGG GTGAAATGG-3′, 5′-CATGTTGGACCACCAACTATTTGG-3′, 5′-CTGATCTCGACAAGTAGTTGTAGG-3′ and 5′-ATGGCGG GAATCCCAGATCATATCC-3′) as well as in seedlings of the wild type, *lht6*, and *lht6*/*LHT6* complementation lines (5′-GCAACGG TTCGATAGGTACC-3′ and 5′-CATCGGATGGTAAACCG TAG-3′). Equal amounts of cDNA per sample were verified by determining expression of the actin gene *ACT2* (At3g18780) (5′-CCAATCGTGTGTGACAATGGTACCG-3′ and 5′-GGTTGT ACGACCACTGGCGTACAAG-3′) (An *et al.*, 1996).

### Histochemical analysis of LHT6 promoter–GUS lines

In previous work, *Arabidopsis* plants carrying an *LHT6* promoter–*GUS* construct were produced (Foster *et al.*, 2008). Six-day old *LHT6* promoter–*GUS* seedlings as well as 2- and 3-week-old *LHT6* promoter–*GUS* plants grown on full-strength MS medium (Murashige and Skoog, 1962) were placed in GUS staining solution containing 2mM X-Gluc substrate (5-bromo-4-chloro-3-indoxyl-β-d-glucuronide; cyclohexyl ammonium salt; Gold Biotechnology, St. Louis, MO, USA), 10mM EDTA (ethylenediaminetetraacetic acid, pH 8), 1mM potassium ferrocyanide, 1mM potassium ferricyanide, 0.1% (w/v) Triton X-100, and 100mM PO_4_ buffer (pH 7), followed by vacuum infiltration for 15min. The seedlings and organs were then incubated overnight at 37 °C and subsequently cleared of chlorophyll with multiple exchanges of 95% (v/v) ethanol. Some tissue samples were embedded in London Resin White Acrylic (Ted Pella Inc., Redding, CA, USA) according to Pélissier *et al*. (2004) and sectioned using a Reichert Ultracut R microtome (Leica, Vienna, Austria). Whole seedlings and plants were analysed with a stereoscopic light microscope (Wild, HeerBrugg, Switzerland), while root sections were viewed with a compound light microscope (Leitz, Wetzlar, Germany).

### Identification of homozygous T-DNA insertion lines

The *LHT6* T-DNA insertion line (SALK_049092.50.70) was obtained from the Arabidopsis Biological Resource Center (Ohio State University, Columbus, OH, USA). This line was screened for homozygous T-DNA insertion by PCR using the primers: T-DNA-specific 5′-TGGTTCACGTAGTGGGCCATCG-3′ and *LHT6*-specific 5′-CTTAAGTGCACTGGGTGAAATGG-3′ and 5′-CAGCACAAGCCCAAAAATGATGC-3′. In previous studies on the AAP1 transporter, *AAP1* T-DNA insertion lines in the Wassilewskija ecotype background were analysed (Lee *et al.*, 2007). To be able to compare *lht6* and *aap1* mutants and for double mutant production, a homozygous *AAP1* T-DNA insertion line of ecotype Columbia (SAIL_871_C03) was identified by PCR using T-DNA-specific 5′-TTCATAACCAATTCTCGATACAC-3′, and *AAP1*-specific 5′-ATGGTCGAGAGAAGCGTACC-3′ and 5′-GATGCAAAACAGGACTGTCG-3′ primers. The PCR products obtained by using gene-specific and T-DNA left border (LB) primers were cloned into the vector pGEM^®^-T Easy (Promega, Madison, WI, USA) and sequenced to determine the exact location of the T-DNA insertions in LHT1 and AAP1, respectively.

### Construct preparation

The *LHT6* promoter and cDNA were isolated and each cloned into pGEM^®^-T Easy as described in Foster *et al.* (2008). To build an *LHT6* promoter–*LHT6* cDNA construct for complementation of the *Arabidopsis lht6* mutant, the *LHT6* cDNA was excised with *Not*I, blunted, and cloned into the blunted *Spe*I site of the pGEM^®^-T Easy vector carrying the *LHT6* promoter (Foster *et al.*, 2008). The *LHT6* promoter–*LHT6* cDNA cassette was then cut with *Nco*I (blunted)/*Pst*I and transferred into the *Sma*I/*Pst*I site of the binary vector pTKAN derived from pPZP212 (Hajdukiewicz *et al.*, 1994). The vector was kindly provided by Karin Schumacher, ZMBP, Tübingen, Germany. The *LHT6* promoter–*LHT6* cDNA in pTKAN was transferred into *Agrobacterium tumefaciens* strain GV3101 (pMP90) (Holsters *et al*., 1980; Koncz and Schell, 1986) and used for transformation of the *lht6* mutant line via the floral dip method (Clough and Bent, 1998).

### Uptake of ^14^C-labelled amino acids

Uptake studies with amino acids at concentrations of 150 μM and 2mM were performed with plants grown horizontally on solid full-strength MS medium (Murashige and Skoog, 1962; pH 5.5–5.7) with myo-inositol (100mg l^–1^), sucrose (10g l^–1^), 2-(*N*-morpholino)-ethane sulphonic acid (MES; 0.5g l^–1^), and agar (8g l^–1^) as described in Lee *et al.* (2007). After 6 d of growth, when the cotyledons/leaves of the seedlings have no contact with the media surface, channels were cut into the solid media and filled with radiolabelled [^14^C]amino acids (Moravek Biochemicals) at 2 μCi along with 5ml of non-labelled amino acids diluted in 2.5mM MES buffer (pH 5.5–5.7) at final concentrations of 150 μM and 2mM, respectively. At least 15 single seedlings were collected after 48h of feeding, rinsed four times in water, and placed in vials with 2ml of scintillation solution containing sample solubilizer (ScintiSafe™ Plus 50% Cocktail, Fisher Chemical). Radioactivity was measured for single seedlings using a Packard Tri-Carb series 1500 liquid scintillation analyzer (Downers Grove, IL, USA). As no differences in seedling dry weight were detected (see Fig. 3A), the total counts per minute and seedling were normalized to the wild type (100%). The results are presented as means ±standard deviation (SD). One-way analysis of variance (ANOVA) was used to determine statistical significance using SigmaPlot 8.0. Each experiment was repeated at least three times. To justify normalization, it was confirmed in three independent experiments that there were no differences in growth between the wild type and mutants by determining fresh and dry weights of whole seedlings by measuring six pools each consisting of eight seedlings.

Uptake of amino acids at concentrations of 30 μM was performed with plants grown vertically for 2 weeks on half-strength glycine-free solid MS medium (pH 5.7), myo-inositol (50mg l^–1^), sucrose (5g l^–1^), MES (0.25g l^–1^), and agar (8g l^–1^). Glycine was removed to exclude potential regulatory effects on uptake of amino acids offered at low concentrations. Plants of each genotype were transferred to plates that were 10mm high and 3.5cm in diameter, and the roots were submerged in 4ml of uptake solution (half-strength liquid MS without N at pH 5.7) containing ^14^C-labelled amino acids at 0.28 μCi and non-labelled amino acids at concentrations of 30 μM. Plants were removed after 3h, rinsed four times in water, and patted dry. Roots and shoots were separated and their fresh weight was analysed for at least five pools of each eight plants. After drying overnight at 60 °C, the root and shoot dry weights were measured for each pool and the radioactivity of the different tissues was determined using liquid scintillation counting (see above). Since no differences in root and shoot dry weight was detected (see Fig. 3B), the total counts per minute could be related to the dry weight of roots or whole plants and were then normalized to the wild type (100%). At least three independently grown sets of plants were analysed.

### *Growth of* Arabidopsis *plants on toxic amino acid analogues*

Wild-type plants, *lht6*, *aap1*, and *lht6/aap1* double mutants were cultured for 21 d on half-strength MS medium (pH 5.7, Murashige and Skoog, 1962) with myo-inositol (50mg l^–1^), sucrose (5g l^–1^), MES (0.25g l^–1^), and agar (8g l^–1^), and containing amino acid analogues at concentrations toxic for the wild type. Specifically, toxic analogues for glutamine [1 μM 2-amino-6-diazo-5-oxo-l-norleucine (DON); Poppenberger *et al.*, 2003], isoleucine [5 μM *o*-menthylthreonine (OMT); Mourad and King, 1995], phenylalanine [70 μM *p*-fluorophenylalanine (FPA); Yan *et al.*, 2000], proline [70 μM azetidine-2-carboxylate (AZC); Hare *et al.*, 2001], glutamate (1 μM *N*-methyl sulphoximine (MSX); Su *et al.*, 2004; Yang *et al.*, 2010), and arginine [50 μM canavanine (CAN); Yan *et al.*, 2000] were used. The growth studies were repeated at least three times and imaged. The fresh and dry weights of roots, shoots, and whole plants were determined in three experiments by measuring six pools each consisting of the tissues of eight plants.

## Results

### LHT6 *is highly expressed in* Arabidopsis *roots and seedlings*

**Fig. 1.**
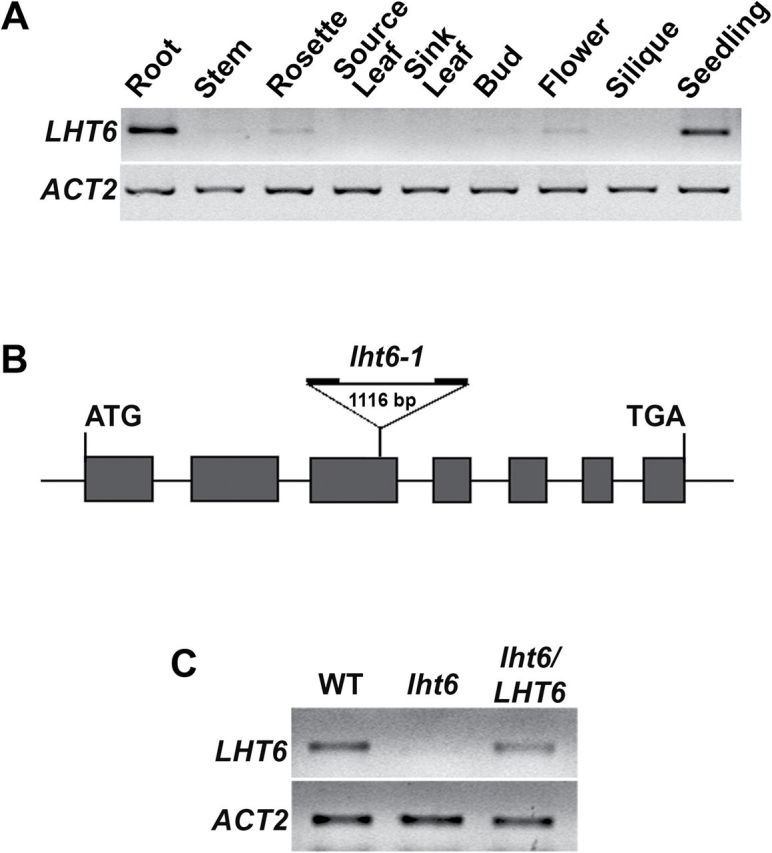
Molecular analyses. (A) Organ-specific expression analysis of *LHT6* by RT–PCR using RNA from 6-day-old seedlings and different plant organs of 6-week-old *Arabidopsis* plants. *AtACT2* expression was used as a control for equal concentrations of cDNA. (B) Schematic diagram of the T-DNA insertion within the *LHT6* gene (SALK 049092). The boxes represent exons, and lines denote introns. (C) *AtLHT6* and *AtACT2* expression analysis by RT–PCR using RNA from seedlings of the wild type (WT), the *lht6* mutant, and the *lht6*/*LHT6* complementation line.

Previous research demonstrated that *LHT6* is expressed in buds and flowers and localized to the plasma membrane (Foster *et al.*, 2008). To determine if the transporter is also present in roots and other organs, reverse transcription–PCR was performed using RNA from different *Arabidopsis* organs of 6-week-old plants and from 6-day-old seedlings. The expression profile showed high levels of *LHT6* transcripts in roots and seedlings, while relatively weak expression was detected in stems, rosette leaves, buds, and flowers (Fig. 1A).

**Fig. 2.**
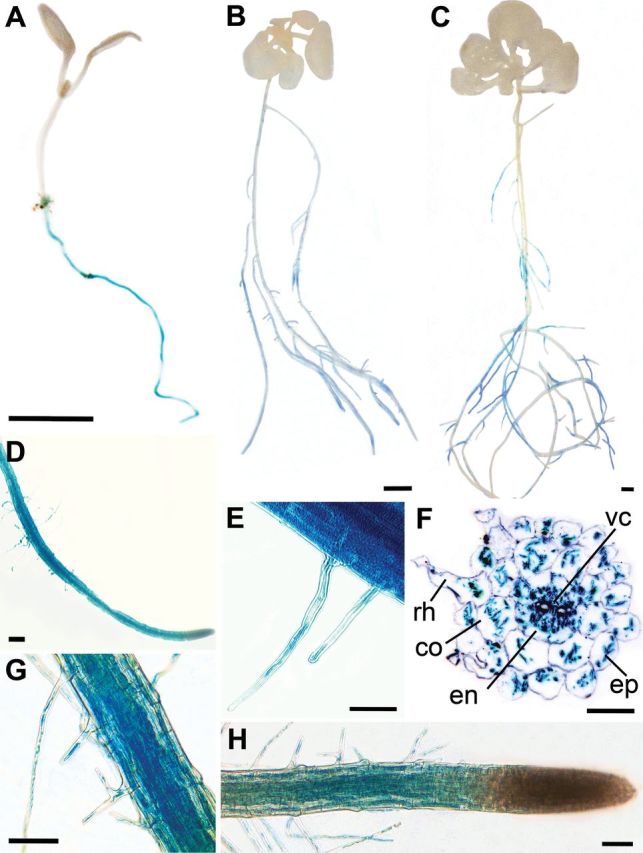
*LHT6* promoter–*GUS* analysis in *Arabidopsis*. GUS staining was observed in the tap root of 6-day-old seedlings (A and D), and in the lateral roots of 2-week-old (B) and 3-week-old (C) plants grown on full-strength MS media. GUS expression was further found in root hairs (E, G and H) and all other cells of 6-day-old seedling roots (F) and of lateral root of 2-week-old (and 3-week-old) plants (G). GUS staining was generally not found in the root tip of 2-week-old (and 3-week-old) plants (H). (F) Inverted dark-field image of a cross-section of a GUS-stained seedling root. The blue colour indicates GUS staining in the different root cells. Lines point to the specific root cells, which are: c, cortex; en, endodermis; ep, epidermis; rh, root hair; vc, cells of the vascular cylinder. Scale bars=1mM (A–C), 100 μm (D), 50 μm (E, G, and H), and 25 μm (F).

To examine tissue-specific localization of *LHT6*, histochemical analysis of *LHT6* promoter–*GUS* lines was performed. GUS staining was found in the taproot of 6-day-old seedlings (Fig. 2A, D) and in lateral roots of 2- and 3-week-old plants (Fig. 2B, C), which is consistent with the high levels of *LHT6* RNA expression in these organs. Within roots, *LHT6* is localized in root hairs (Fig. 2D–H) as well as in other root cells involved in uptake including epidermal, cortex, and endodermis cells of seedlings and growing plants (Fig. 2F, G). GUS staining was generally not found in the root tips of 2- and 3-week-old plants (Fig. 2H).

### LHT6 *expression is knocked out in the mutant*

To analyse LHT6 function *in planta*, a homozygous *Arabidopsis LHT6* T-DNA insertion line (*lht6*; SALK_049092.50.70) was isolated containing a T-DNA in the third out of seven exons of the *LHT6* gene (Fig. 1B). Further, *lht6/LHT6* complementation lines were produced by transforming the *lht6* plants with an *LHT6* promoter–*LHT6* cDNA construct. *LHT6* transcript levels were analysed in 2-week-old wild-type, *lht6*, and *lht6*/*LHT6* plants by RT–PCR. The results showed similar levels of *LHT6* expression in wild-type and *lht6*/*LHT6* plants, whereas no transcripts were detected in the *lht6* mutant (Fig. 1C). This suggests that LHT6 function is knocked out in the mutant, while transporter expression and function is restored in the complementation line.

### Growth analyses of mutants

**Fig. 3.**
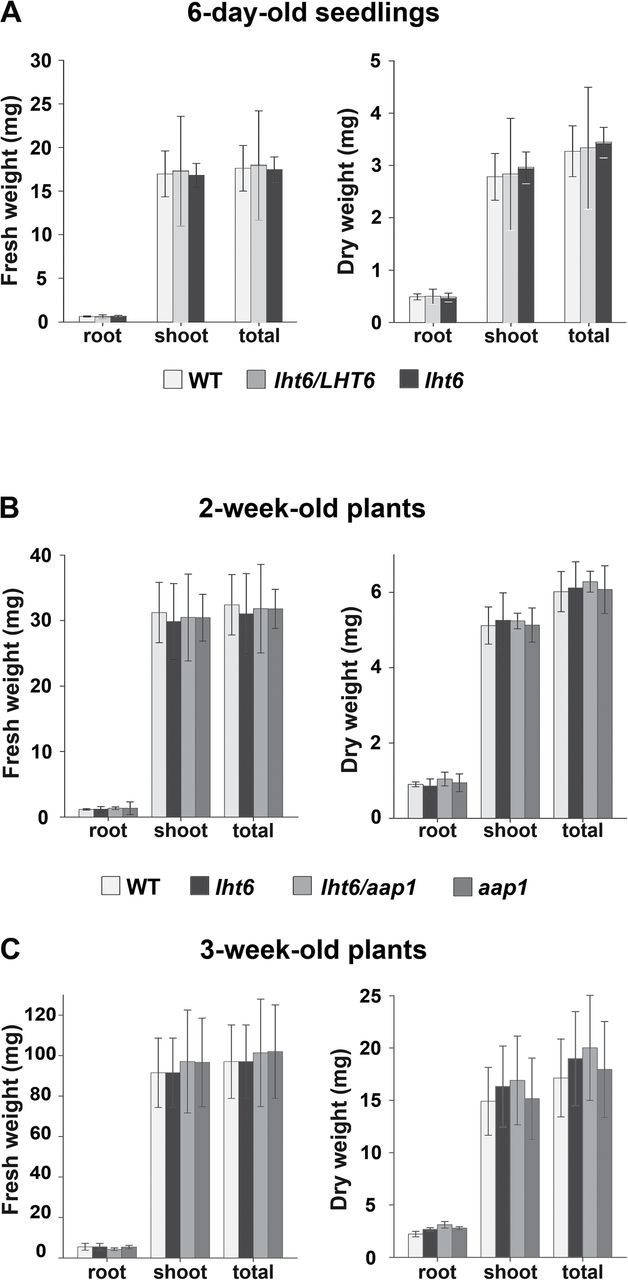
Fresh and dry weights of roots, shoots, and whole plants (total) of *lht6*, *lht6/LHT6*, *aap1*, *lht6/aap1*, and the wild type grown on media. (A) Six-day-old seedlings. (B) Two-week-old plants. (C) Three-week-old plants. Plants were grown on full-strength MS medium (A) and half-strength MS medium (B and C). Fresh and dry weights of roots, shoots, and whole plants were determined by measuring six pools (*n*=6) each consisting of the tissues of eight plants. Results are shown for one experiment, but three independent experiments were performed. Error bars depict the standard deviation (± SD). Asterisks indicate significant differences when using one-way analysis of variance (ANOVA; *P*-values ≤0.05).

**Fig. 4.**
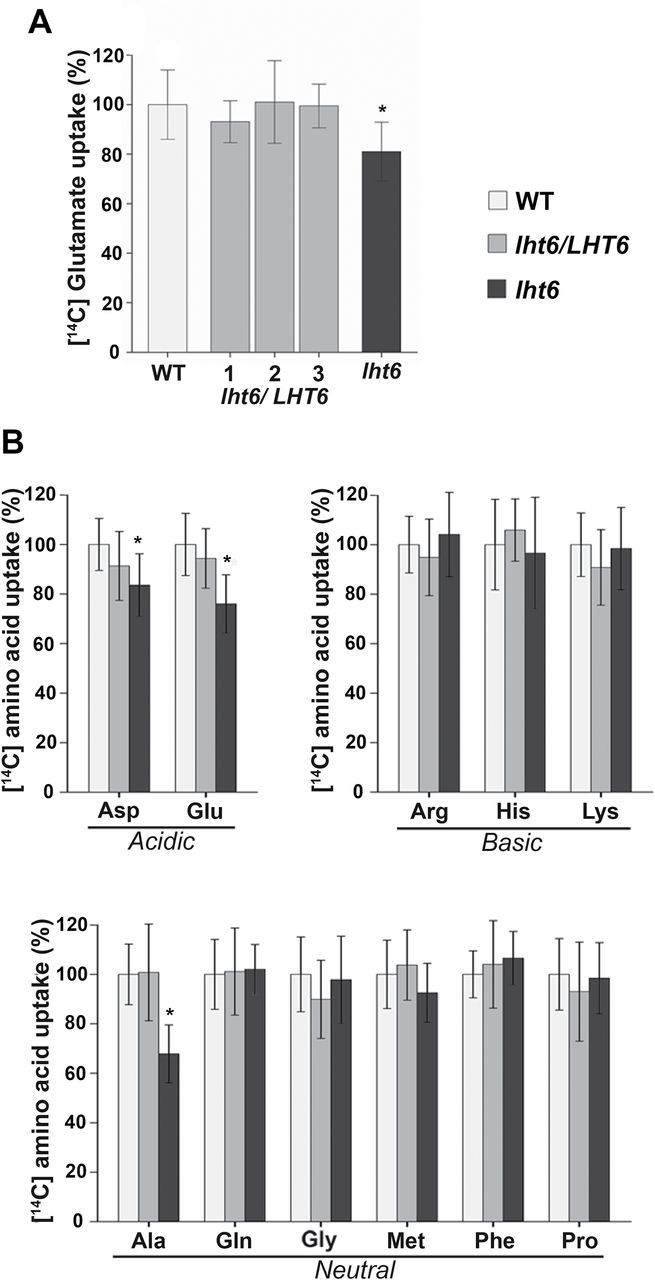
Uptake of ^14^C-labelled amino acids by 6-day-old *lht6*, *lht6/LHT6*, and wild-type (WT) seedlings exposed to 2mM amino acid concentrations. (A) Uptake studies using [^14^C]glutamate and plants from the WT, *lht6* mutant, and three *lht6/LHT6* complementation lines. (B) Uptake studies using acidic, basic, and neutral amino acids. Since no differences in fresh or dry weights were detected for *lht6*, *lht6/LHT6*, and WT seedlings (see Fig. 3A), the total counts per minute and per seedling could be normalized to the WT, which was set to 100%. Error bars depict the standard deviation (± SD). Asterisks indicate significant differences when using one-way analysis of variance (ANOVA; *P*-values ≤0.05). Results are shown for one experiment and are a mean of a minimum of 15 replicates (*n≥*15). Three independent experiments were performed.

To analyse if the mutations in the *LHT6* or *AAP1* transporter affect root or shoot growth of mutants versus the wild type when cultured on media, root, shoot, and whole-plant fresh and dry weights of 6-day-old seedlings as well as of 2- and 3-week-old plants were determined. The results demonstrate that when knocking the transporters out or down, growth of *lht6*, *aap1*, and *lht6/aap1* double mutants is not affected (Fig. 3). This result will also help to determine if the observed differences in amino acid uptake are in fact due to changes in transport activities rather than to alterations in growth or biomass. It is noteworthy that in some cases (see Figs 4 and 7A), biomass and uptake studies were performed with different sets of plants, and there might be the possibility of variation.

### *Uptake of alanine, aspartate, and glutamate offered at high concentrations is decreased in* lht6 *plants*

To resolve if LHT6 is functioning in amino acid acquisition at high concentrations, growth and uptake studies were performed with *lht6*, wild-type, and *lht6*/*LHT6* plants (cf. Lee *et al.*, 2007). While no differences were observed when plants were grown on media supplemented with single amino acids, uptake studies with ^14^C-labelled amino acids (2mM) revealed changes in amino acid acquisition between mutant and the wild type (Fig. 4). First, uptake of [^14^C]glutamate was analysed since this amino acid only slightly, if at all, inhibits growth of *Arabidopsis* plants even when it is offered at very high concentrations (Lee *et al.*, 2007). In addition, three *lht6/LHT6* lines were tested to determine if in all lines LHT6 function is fully restored and if they perform like the wild type (Fig. 4A). The results show that uptake of glutamate was significantly decreased by ~19% in the *lht6* mutant compared with the wild-type and *lht6/LHT6* plants. No difference was found in glutamate root uptake between the wild type and the complementation lines (Fig. 4A). These data support that the observed reduction in glutamate uptake for the *lht6* mutant is in fact due to the knockout of *LHT6* and that LHT6 is involved in glutamate uptake when offered in high amounts (Fig. 4). Uptake of aspartate, and neutral and basic ^14^C-labelled amino acids was then analysed. The results demonstrate that in addition to glutamate, aspartate and alanine uptake are also reduced in the *lht6* mutant versus the wild type or complementation line by 17% and 32%, respectively (Fig. 4B). This shows that at high amino acid concentration LHT6 transports acidic amino acids and alanine.

### *Growth of* aap1 *and* lht6/aap1 *plants is affected on low levels of toxic amino acid analogues*

**Fig. 5.**
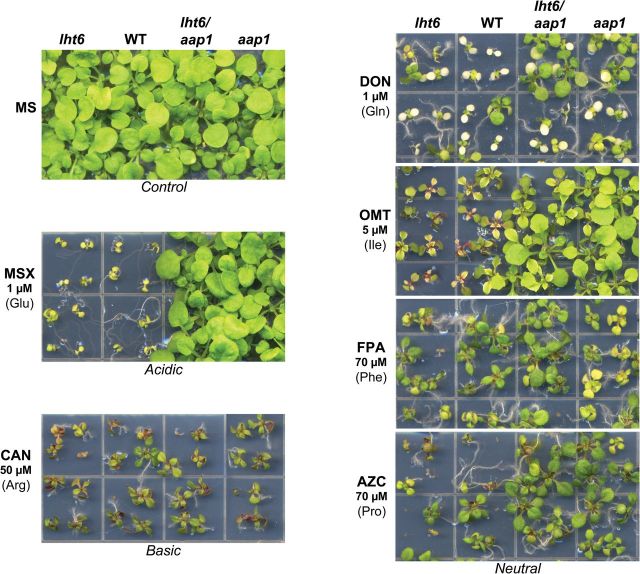
Growth of wild-type (WT), *lht6*, *aap1*, and *lht6/aap1* plants on toxic amino acid analogues. Plants were grown for 3 weeks on half-strength MS medium containing low concentrations (μM) of the amino acid analogue inhibiting growth of WT plants. Analogues for glutamate (*N*-methyl sulfoximine; MSX), arginine (canavanine; CAN), glutamine (2-amino-6-diazo-5-oxo-l-norleucine; DON), isoleucine (*O*-menthy-l-threonine; OMT), phenylalanine (fluorophenylalanine; FPA), and proline (azetidine-2-carboxylate; AZC) were tested. The growth studies were repeated at least three times. (This figure is available in colour at *JXB* online.)

**Fig. 6.**
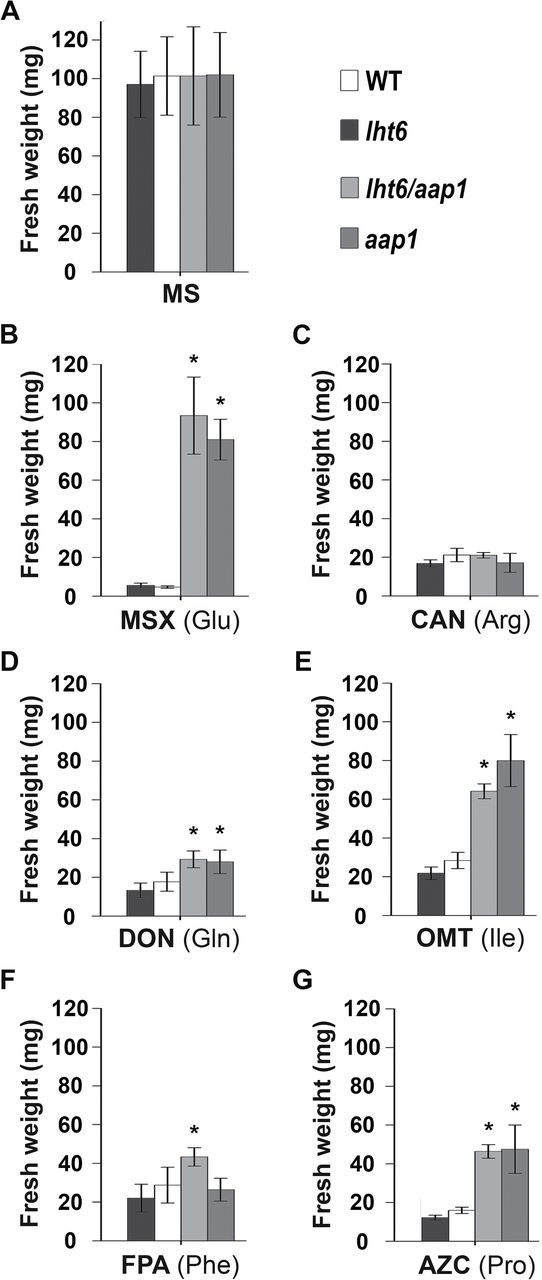
Fresh weight of wild-type (WT), *lht6*, *aap1*, and *lht6/aap1* plants grown on media with toxic amino acid analogues (see Fig. 5). Plants were grown for 3 weeks on half-strength MS medium containing low concentrations of the amino acid analogue inhibiting growth of WT plants. Analogues for glutamate (1 μM *N*-methyl sulfoximine; MSX), arginine (50 μM canavanine; CAN), glutamine (1 μM 2-amino-6-diazo-5-oxo-l-norleucine; DON), isoleucine (5 μM *O*-menthy-l-threonine; OMT), phenylalanine (70 μM fluorophenylalanine; FPA), and proline (70 μM azetidine-2-carboxylate; AZC) were tested. Fresh and dry weights were determined by measuring six pools (*n*=6) each containing eight plants. Results are shown for one experiment, but three independent experiments were performed. Error bars depict the standard deviation (± SD). Asterisks indicate significant differences when using one-way analysis of variance (ANOVA; *P*-values ≤0.05).

To resolve if LHT6 plays a role in amino acid uptake at ecological concentrations, wild-type and *lht6* plants were analysed for their ability to germinate and grow on media containing low levels of amino acid analogues that are toxic to wild-type plants. Specifically, toxic analogues for glutamine (DON), isoleucine (OMT), phenylalanine (FPA), proline (AZC), glutamate (MSX), and arginine (CAN) were used (Figs 5 and 6). In addition, growth of the *aap1* mutant and *lht6*/*aap1* double mutant was analysed. AAP1 has previously been shown to play a role in uptake of amino acids by the root, at least when amino acids are offered at high concentrations (Lee *et al.*, 2007; Svennerstam *et al.*, 2011). The *lht6* mutant showed no differences in growth compared with the wild type on any of the toxic analogues tested (Figs 5 and 6). However, the *aap1* and *lht6/aap1* plants grew better than wild-type and *lht6* plants on media with toxic analogues for glutamate, isoleucine, proline, and glutamine (Figs 5 and 6). Dependent on the amino acid analogue, fresh weights were 50–190% higher in *aap1* and *lht6/aap1* compared with wild-type or *lht6* plants (Fig. 6B, D, E, G). This suggests that less of the organic N compounds were taken up due to the knockout of *AAP1* expression. Additionally, the *lht6/aap1* plants grow ~50% better than the single mutants and wild type on the phenylalanine analogue (Figs 5 and 6F). This points to some function of both LHT6 and AAP1 in phenylalanine transport that is only seen in the double mutant. Neither the single nor the double mutants showed a change in growth on the arginine analogue, indicating that the basic amino acid is not a substrate for LHT6 or AAP1 (Figs 5 and 6C). Together, these results suggest a role for AAP1 in the uptake of glutamate and neutral amino acids, when they are present at low concentrations in the growth media. The lack of a growth phenotype for *lht6* mutants might suggest that (i) the analogues/amino acids tested are not substrates for LHT6 and/or (ii) other root amino acid transporters (e.g. AAP1 and LHT1) compensate for LHT6 function. However, while growth studies on toxic analogues are useful to draw conclusions on transporter function in uptake of a specific physiological substrate, they need also be interpreted with care, since inhibitory growth effects might be dependent on the specific analogue rather than its concentration. As shown in this study, for example, MSX and DON are already toxic for the wild type at a low level (1 μM) while OMT or FPA inhibit growth at 5 μM or 70 μM, respectively (Figs 5 and 6). In addition, the affinity of a transporter for the amino acid versus its analogue might differ (cf. Mourad and King, 1995). Therefore, to resolve LHT6 and AAP1 function in amino acid import into *Arabidopsis* roots further, uptake studies were performed at low amino acid concentrations.

**Fig. 7.**
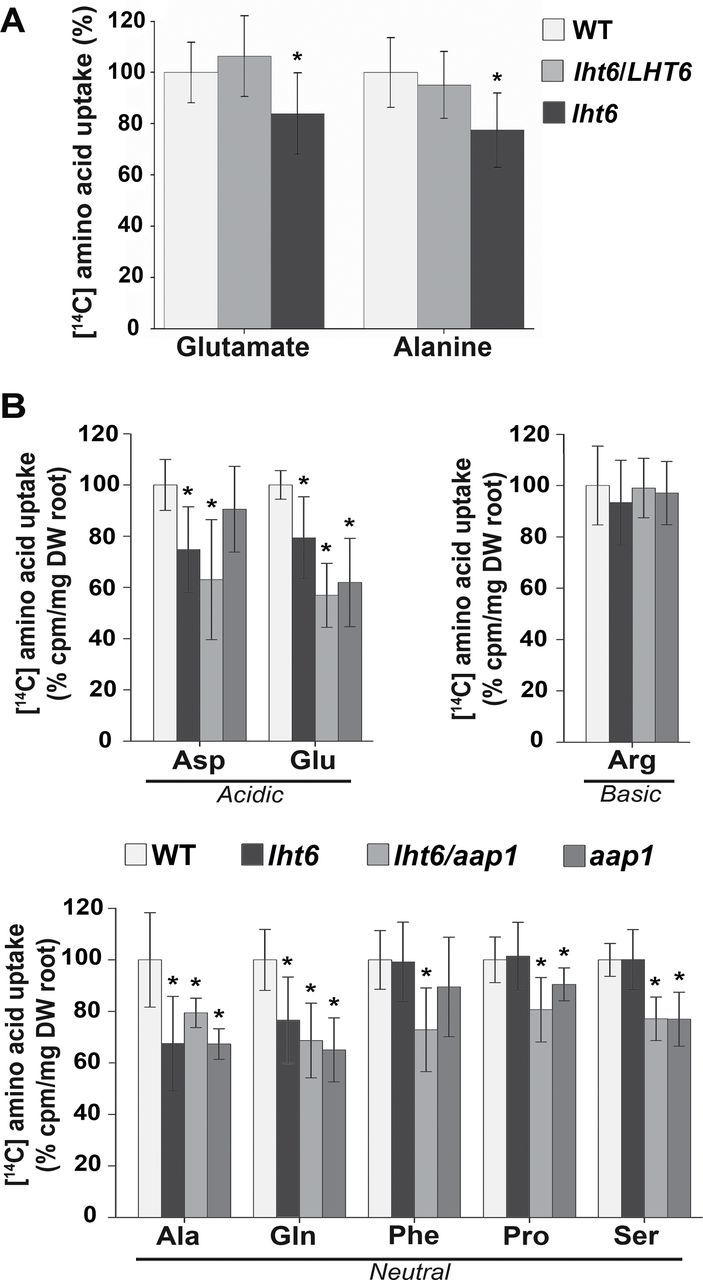
Uptake studies using amino acids at soil concentrations. (A) Uptake of [^14^C]glutamate and [^14^C]alanine at 150 μM concentrations by 6-day-old wild-type (WT), *lht6*, and *lht6/LHT6* seedlings. Studies were performed with at least 15 seedlings (*n≥*15). As no differences were detected in seedling dry weight (see Fig. 3A), radioactivity was measured for single seedlings, and the total counts per minute and seedling were normalized to the WT type, which was set to 100%. (B) Uptake of ^14^C-labelled acidic, basic, and neutral amino acids at 30 μM concentrations by roots of roots of 2-week-old WT, *lht6*, *aap1*, and *lht6/aap1* plants. Five pools of plants (*n*=5) were analysed each containing eight plants. Since no differences in root and shoot dry weight were detected (see Fig. 3B, C), the total counts per minute could be related to dry weight of roots and were then normalized to the WT (100%). Results are shown for one experiment, but three independent experiments were performed. Error bars depict the standard deviation (± SD). Asterisks indicate significant differences when using one-way analysis of variance (ANOVA; *P*-values ≤ 0.05).

### *Uptake of neutral and acidic amino acids offered at biologically relevant concentrations is reduced in* lht6, aap1, *and* lht6/aap1 *plants*

Uptake studies were performed to analyse the function of the amino acid transporters in organic N import into roots at ecological concentrations. Free amino acid concentrations found in the soil solution vary greatly and might range from 0 to 150 μM (Chapin *et al.*, 1993; Raab *et al.*, 1996, 1999). In previous work, it was demonstrated that AAP1 is involved in root uptake of ^14^C-labelled alanine and glutamine at concentrations of 150 μM (Lee *et al.*, 2007). Here, it was tested if LHT6 might have a similar or diverse function. Uptake studies were performed with 6-day-old seedlings that developed a taproot with root hairs (see Fig. 2A, D–F). The results show a decrease in both [^14^C]alanine and [^14^C]glutamate acquisition in *lht6* compared with wild-type or *lht6/LHT6* seedlings of 23% and 16%, respectively (Fig. 7A).

Further, uptake studies using ^14^C-labelled neutral, acidic, and basic amino acids at concentrations of 30 μM were performed with 2-week-old *lht6*, *aap1*, and *lht6/aap1* plants that had developed lateral roots (see Fig. 2B, G, H). Uptake was measured as total counts per minute and related to root dry weight (Fig. 7B) and whole plant dry weight (Supplementary Fig. S1 available at *JXB* online) and they were then normalized to the wild type (100%). The *lht6* plants showed a significant reduction between 20% and 27% in [^14^C]alanine, [^14^C]glutamine, [^14^C]aspartate, and [^14^C]glutamate root uptake, respectively, but no change in acquisition of basic and other neutral amino acids compared with the wild type (Fig. 7B). For *aap1* and *lht6/aap1* plants, a significant decrease was observed in root uptake of alanine, glutamine, proline, serine, and glutamate that, dependent on the amino acid, ranged from 10% to 43%. In addition, the double mutant displayed reduced uptake of phenylalanine. During the uptake studies, some of the labelled amino acids (4–28% dependent on the amino acid) are already translocated in the xylem to the transpiring leaves. Therefore, whole seedling uptake was also determined (Supplementary Fig. S1). The results were generally consistent with root uptake data, showing differences for alanine, glutamine, proline, serine, and glutamate (compare Fig. 7B with Supplementary Fig. S1).

## Discussion

### LHT6 functions in uptake of amino acids from the environment

Five *Arabidopsis* transporters have recently been identified that might play a role in uptake of a broad spectrum of amino acids from the soil, AAP1, LHT1, AAP5, CAT6, and CAT8 (for a review, see Tegeder, 2012). LHT1 (Hirner *et al.*, 2006), CAT6 (Hammes *et al.*, 2006), and CAT8 (Yang *et al.*, 2010) seem to function in amino acid import into the root tips of developing *Arabidopsis* plants, while the location of AAP5 function has not been resolved. Only AAP1 was found in root cells that are predicted to play a major role in nutrient uptake from the soil, including epidermis cells and root hairs (Dittmer, 1937; Itoh and Barber, 1983; Lazof *et al.*, 1992, 1996; Gassmann and Schroeder, 1994; Gahoonia and Nielsen 1998; Lee *et al.*, 2007). In this study, *LHT6* was shown to be expressed in the same root cell types as AAP1, suggesting a role for LHT6 in N acquisition from the environment (cf. Lee and Tegeder, 2004 and Fig. 2; see Fig. 7B). LHT6 is a member of the LHT-like family containing high affinity transport systems for acidic and neutral amino acids (Lee and Tegeder, 2004; Hirner *et al.*, 2006; Svennerstam *et al*., 2007, 2011). In fact, feeding experiments with labelled amino acids demonstrated that LHT6 imports amino acids into the root, specifically acidic amino acids, alanine and glutamine, at low concentrations, but also acidic amino acids and alanine when offered at high amounts (see Figs 4 and 7; Supplementary S1 at *JXB* online).

### LHT6 and AAP1 display different as well as overlapping functions

Previous uptake studies with *aap1* mutants suggested that AAP1 is involved in glutamate and neutral amino acid import when available at high concentrations (Lee *et al.*, 2007; Svennerstam *et al.*, 2011). However, growth studies using toxic amino acid analogues at very low concentrations showed that *aap1* compared with wild-type plants grew better on analogues for glutamate, glutamine, isoleucine, and proline (Figs 5 and 6). This suggests that less of the toxic analogues were taken up by the mutant, and that AAP1 might import glutamate and selective neutral amino acids into root cells when present at low levels in the medium. Uptake studies corroborated these predictions (Fig. 7B) and demonstrated AAP1-mediated import of alanine, glutamine, proline, serine, and glutamate into the root at naturally occurring soil concentrations. However, basic amino acids (arginine) seem not to be significant substrates for AAP1 (and LHT6) at these low concentrations. This is, at least in part, in contrast to recent studies by Svennerstam *et al.* (2011) who performed similar experiments with a different *aap1* knockout mutant with T-DNA insertion (GABI-KAT 135G05; see http://www.gabi-Kat.de, last accessed 20 June 2014) and using a low level of glutamine, alanine, aspartate, glutamate, arginine, and lysine. They could not observe any significant differences in uptake of alanine, glutamine, and glutamate between *aap1* mutants and the wild type, while uptake of arginine and lysine seemed increased. Future experiments with the GABI-KAT *aap1* mutant (Svennerstam *et al.* 2011), such as growth studies on toxic amino acid analogues, might help to resolve this inconsistency. The observed phenotype associated with the growth and uptake studies for the *aap1* and *lht6/aap1* double mutants but not for the *lht6* mutant also indicates that the transport function of AAP1 at low levels of glutamate, glutamine, alanine, isoleucine, proline, and serine is not compensated for by LHT6.

In contrast, the *lht6* mutant compared with the wild type showed no differences in either growth or uptake of the majority of neutral amino acids tested (Figs 4–7), suggesting that LHT6 might simply not transport these organic N forms. However, it cannot be excluded that AAP1 and/or LHT1 are compensating for LHT6 function when the transporter is knocked out. On the other hand, alanine and amides, specifically glutamine, are transported by LHT6 as uptake is decreased when this amino acid is offered in low amounts. Both *lht6* and *aap1* seem to mediate some uptake of phenylalanine at very low concentrations as the double mutant shows a significant decrease in phenylalanine acquisition (Fig. 7).

In the case of acidic amino acids, both transporters import glutamate into the root cells, when supplied at ecologically relevant concentrations, as indicated by a significantly reduced uptake of the charged amino acid in both single *lht6* and *aap1* mutants and double mutant (see Fig. 7; Lee *et al.*, 2007). However, aspartate seems to be only transported by LHT6. Previous studies using heterologous expression analysis led to the conclusion that members of the LHT family are high affinity transport systems for aspartate (Lee *et al.*, 2004; Hirner *et al.*, 2006), which is consistent with the observation that aspartate uptake in the *lht6* mutant was reduced by 25% when the amino acid was supplied at 30 μM. This is further in agreement with the observed reduction in aspartate acquisition in the double mutant, as well as with the lack of AAP1 function in aspartate transport when the transporter was biochemically analysed in yeast, *Xenopus* oocytes, and plants (Fig. 7; Fischer *et al*., 1995, 2002; Lee *et al.*, 2004; Hirner *et al.*, 2006; Svennerstam *et al*., 2008, 2011). Taken together, the growth and uptake studies support that when amino acids are present at low concentrations (i) LHT6 takes up acidic amino acids, alanine and glutamine, into the root cells; and (ii) AAP1 transports neutral amino acids and glutamate. However, additional amino acids might be substrates for the respective transporters and/or the lack of *LHT6* or *AAP1* expression might be counter-balanced by other amino acid transporters present in root hairs and epidermal cells.

### Importance of root amino acid uptake systems and their regulation

Studies in maize (and other plant species) analysing uptake via root tips versus upper root parts support that the majority of amino acids are taken up via the root epidermis and hairs (Dittmer, 1937; Itoh and Barber, 1983; Lazof *et al.*, 1992, 1996; Gassmann and Schroeder, 1994; Gahoonia and Nielsen 1998), and LHT6 and AAP1 are localized to the plasma membrane of these cell types (Fig. 2; Lee *et al.*, 2007; Foster *et al.*, 2008). Thus, surprisingly, in *lht6* and *aap1* single or double mutants only a 10–43% reduction in uptake was observed for those amino acids that are substrates for the LHT6 and AAP1 transporters (Fig. 7). This might be due to the presence of other, as yet unidentified transporters that are also expressed in similar cells, such as members of the CAT family (Hammes *et al.*, 2006; Yang *et al.*, 2010). In addition, it might well be that in *Arabidopsis* both the symplasmic and the apoplasmic transport routes are of high relevance for amino acid uptake into root cells. The apoplasmic route would require amino acid importers in the cortex and endodermis cells to circumvent the apoplasmic blockade (i.e. Casparian strip) and to move the organic N to the vascular bundle. At least in root nodules, such cortex- and endodermis-localized organic N transporters have recently been found, and their importance for shoot N supply has been demonstrated (Collier and Tegeder, 2012). *LHT6* is not only expressed in the root epidermis and root hairs, but also in the cortex and endodermis, supporting a function in both the symplasmic and apoplasmic transport pathway. These transport pathways might further involve other transporters including AAP5 for basic amino acids, as *aap5* mutants show a severe reduction in arginine and lysine uptake (Svennerstam *et al*., 2008, 2011), and root cell sorting experiments with young seedlings indicate expression of *AAP5* in the root cortex and endodermis (Brady *et al.*, 2007; Gifford *et al.*, 2008). However, localization of AAP5 function in roots of seedlings and especially in developing plants still remains to be resolved. In addition, uptake of amino acids by the root tip may play a more important role than previously assumed, since mutants of *LHT1* show a reduction in uptake of neutral and acidic amino acids by up to 80% and the expression of *LHT1* in developing plants seems to be constrained to the tip of lateral roots (Hirner *et al.*, 2006; Svennerstam *et al*., 2007, 2008, 2011).

On the other hand, the strong reduction in root amino acid acquisition in *lht1* plants might also hint at an additional, regulatory role for LHT1 in amino-N uptake. In the present studies, uptake experiments were performed in 2-week-old plants when *LHT6* and *AAP1* expression in the wild type is restricted to the root (see Fig. 2; Lee *et al.*, 2007) while uptake studies with *lht1* mutants were assayed when wild-type *LHT1* expression is high in mesophyll cells (cf. Hirner *et al.*, 2006; Svennerstam *et al*., 2007, 2011). Further, compared with wild-type plants, *lht1* mutants accumulate amino acids in the leaf cell wall. These high apoplasmic levels might negatively regulate root uptake of neutral and acidic amino acids, finally leading to a severe reduction in amino acid acquisition in the *lht1* plants (Hirner *et al.*, 2006; Forsum *et al.* 2008; Svennerstam *et al*., 2007, 2008, 2011). A similar regulation has been shown for nitrate and ammonium uptake, where high amino acid levels down-regulate N uptake (Wang *et al.*, 2012; Nacry *et al.*, 2013). In line with this is also the observation that N uptake from the soil seems to be positively regulated when amino acid import into leaf cells is increased (Tan *et al.*, 2010; Zhang *et al.*, 2010). This further suggests that, while amino acid transporter function in root cells is generally important for organic N acquisition from the soil, their regulation by shoot-localized transporters such as LHT1 (Hirner *et al.*, 2006; Tan *et al.*, 2010; Zhang *et al.*, 2010) or by shoot-derived signals might be essential to govern the amounts of amino acids that are taken up by the root.

To exclude potential shoot effects on root amino acid uptake, Hirner *et al.* (2006) re-expressed the *LHT1* transporter in the shoot of *lht1* plants by using a leaf-specific promoter. When cultured on high (5mM) aspartate, the root-specific *lht1* mutants showed the same growth phenotype as *lht1* plants. Therefore, it was concluded that LHT1 function in roots rather than in mesophyll cells is responsible for aspartate uptake. Unfortunately, uptake experiments with aspartate or other amino acids were not carried out to support this conclusion further. These would be especially interesting as LHT6 is also involved in root aspartate import at both low and high concentrations (see Figs 4 and 7), and one might expect that loss of LHT1 function in roots is, at least in part, complemented by LHT6. On the other hand, *LHT6* seems not to be expressed in root tips (Fig. 2H), and aspartate uptake might mainly occur via the tip (see above). Clearly, further studies will need to be performed to dissect the role of root-localized transporters in amino acid acquisition versus the regulatory function of shoot-expressed transporters and their contribution to root N uptake.

## Supplementary data

Supplementary data are available at *JXB* online.

Figure S1. Uptake of ^14^C-labelled acidic, basic, and neutral amino acids at 30 μM concentrations by whole (root and shoot) 2-week-old wild-type, *lht6*, *aap1*, and *lht6/aap1* plants.

Supplementary Data

## Abbreviations:

AAP: amino acid permease
AZC: azetidine-2-carboxylate
CAN: canavanine
DON: 2-amino-6-diazo-5-oxo-l-norleucine
FPA: *p*-fluorophenylalanine
LHT: lysine-histidine-like transporter
MSX: *N*-methyl sulphoximine
OMT: *o*-menthylthreonine
ProT: proline transporter
