# Supplementary material for: Different and overlapping functions of Arabidopsis LHT6 and AAP1 transporters in root amino acid uptake
Source: J Exp Bot. 2014 Jul 8;65(18):5193–204. doi: 10.1093/jxb/eru278 (PMC4157705; doi:10.1093/jxb/eru278)
Supplement: Supplementary Data [file supp_65_18_5193__index.html]

Different and overlapping functions of Arabidopsis LHT6 and AAP1 transporters in root amino acid uptake — Different and overlapping functions of Arabidopsis LHT6 and AAP1 transporters in root amino acid uptake — Supplementary Data 

# Different and overlapping functions of *Arabidopsis* LHT6 and AAP1 transporters in root amino acid uptake

## Supplementary Data

Data files

**Files in this Data Supplement:**

- Supplementary Data - Supplementary Data
