# Supplementary material for: Different and overlapping functions of Arabidopsis LHT6 and AAP1 transporters in root amino acid uptake
Source: J Exp Bot. 2014 Jul 8;65(18):5193–204. doi: 10.1093/jxb/eru278 (PMC4157705; doi:10.1093/jxb/eru278)
Supplement: Supplementary Data [file supp_eru278_jexbot117549_file001.pdf]

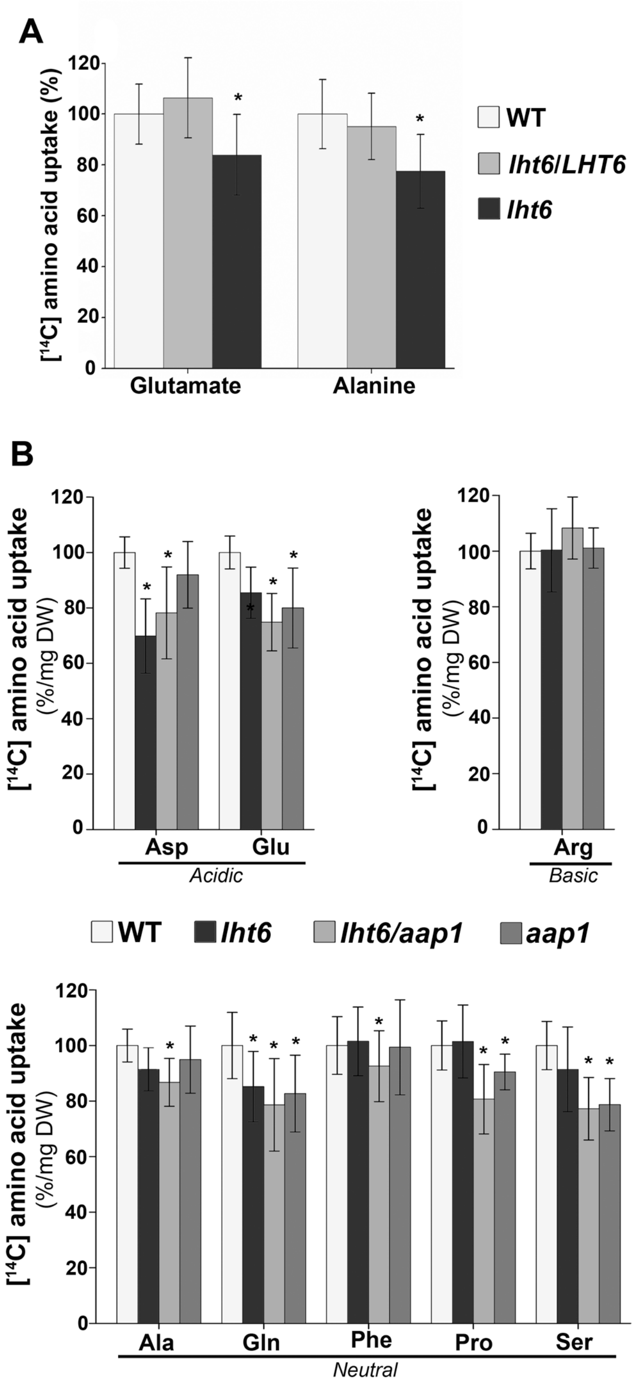

Fig. S1. Uptake of [^{14}C]- labeled acidic, basic and neutral amino acids at 30 µM concentrations by 2-week-old wild-type, *lht6*, *aap1* and *lht6/aap1* plants. Five pools of plants (*n*=5) were analyzed each containing eight plants. Since no differences in root or shoot dry weight were detected (see Fig. 3 A to C), the total counts per minute could be related to dry weights of whole plants, and total

counts per minute per mg dry weight were then normalized to wild-type (100%). Results are shown for one experiment, but three independent experiments were performed. Error bars depict standard deviation (±SD). Asterisks indicate significant differences when using one-way analysis of variance (ANOVA; P values ≤ 0.05).
